# The Telomeric Response to Viral Infection

**DOI:** 10.3390/v9080218

**Published:** 2017-08-09

**Authors:** Zhuo Wang, Zhong Deng, Steve Tutton, Paul M. Lieberman

**Affiliations:** The Wistar Institute, Philadelphia, PA 19104, USA; zwang@wistar.org (Z.W.); dengz@wistar.org (Z.D.); stutton@wistar.org (S.T.)

**Keywords:** TERRA, ALT, telomere, virus, PML-NB, ND10, IFI16, p53, exosomes, innate immunity

## Abstract

The ends of linear genomes, whether viral or cellular, can elicit potent DNA damage and innate immune signals. DNA viruses entering the nucleus share many features with telomeres in their ability to either suppress or co-opt these pathways. Here, we review some of the common mechanisms that viruses and telomeres use to manage the DNA damage and innate immune response pathways. We highlight recent studies on the role of the telomere repeat-containing RNA (TERRA) in response to viral infection. We discuss how TERRA can be activated through a p53-response element embedded in a retrotransposon-like repeat found in human subtelomeres. We consider how TERRA can function as a danger signal when secreted in extracellular vesicles to induce inflammatory cytokines in neighboring cells. These findings suggest that TERRA may be part of the innate immune response to viral infection, and support the hypothesis that telomeres and viruses utilize common mechanisms to maintain genome integrity and regulate innate immunity.

## 1. Introduction

Viruses and telomeres share numerous structural and molecular features that require the competitive utilization of common factors required to propagate and maintain their respective genomes [1]. For the large double-stranded DNA viruses that enter the nucleus, like adenovirus and herpesvirus families, the comparison with telomeres is apparent. Both molecules can appear as double strand breaks and elicit a strong DNA damage response (DDR) if not for other mechanisms that actively suppress the DDR. The relationship between telomeres and small circular DNA viruses (e.g., papilloma and polyomaviruses) or cytoplasmic DNA viruses (e.g., poxviruses), as well as RNA viruses, seems more remote, yet these viruses can share common structures and factors with cellular telomeres [2,3]. The hypothesis that telomeres are derived from selfish DNA or RNA retro-like elements (e.g., group II introns) has been proposed and provokes further consideration of the fundamental similarities between the viruses and telomeres [4]. Telomeres may also provide the host cell with anti-viral functions by trapping invading genomes and suppressing their expression through inaccessible heterochromatic structures. In the broadest terms, viruses and telomeres may be considered selfish genetic elements that share and compete for components of genome propagation and maintenance. In this review, we revisit several studies from our lab and others that highlight the conceptual and mechanistic connections between viruses and telomeres. We consider how these findings are related to other advances in virology and telomere biology, and how they may shed light on our understanding of genome biology and the evolution of genetic systems.

## 2. Telomeres as Functional Genetic Elements

Telomeres are repetitive elements at the ends of linear chromosomes that are essential for maintaining genomic stability [5,6,7]. Telomere repeats are the binding sites for a protective protein complex, termed shelterin, that prevents DNA damage signaling and facilitates the recruitment of telomere-specific replication factors [8,9]. The telomere repeats can be expanded or contracted through dedicated reverse transcriptase telomerase, or by the alternative lengthening of telomeres (ALT) through a homologous recombination process [10,11]. In some organisms, such as drosophila, telomeres can be expanded through retrotransposition [12]. The telomere repeats can be transcribed to generate a non-coding RNA, termed TERRA (telomere repeat-containing RNA), that has been identified in numerous organisms, and contributes structurally and functionally to telomere regulation [13,14,15]. TERRA can be induced in response to various types of stress, including DNA damage and viral infection [16]. TERRA can associate with telomere repeats in *cis* and other genetic loci in *trans* by interacting with a large network of proteins, including epigenetic and telomeric factors [17]. TERRA transcription is regulated by the regions immediately adjacent to telomeres, which are referred to as the subtelomeres [18]. Subtelomere regions are among the fastest evolving regions of the cellular genomes [19]. These regions are highly heterogeneous in populations and vary significantly between species. They are also frequent sites of new virus integration and endogenous retrotransposition. The dynamic maintenance and self-replicating properties of telomeres share many features with viruses, suggesting that they are evolutionarily derived from virus-like elements [4]. 

## 3. Telomeric Chromatin Structure and Conformation 

Nucleosome assembly and chromatin organization are critical for genome maintenance and protection against the various DNA damage sensing and repair pathways. Telomeres have a complex chromatin structure, are typically enriched with heterochromatic features, and are localized to the periphery of the nucleus [20]. The histone H3.3 chaperone complex of Alpha-thalassemia /mental retardation X-linked protein (ATRX) and Death-associated protein (DAXX) is thought to have a special role in assembling chromatin at GC-rich repetitive elements such as telomere repeats [21]. The chromatin structures assembled by ATRX-DAXX-H3.3 may suppress ALT formation at telomeres, as mutations in each of these factors have been linked to ALT-associated tumorigenesis [22,23]. Telomeric non-coding RNA TERRA can facilitate heterochromatin formation through the recruitment of Heterochromatin protein 1 (HP1) and the Origin recognition complex (ORC) [24], as well as numerous other RNA-binding and DDR factors [25,26,27]. Viral non-coding RNAs, such as Herpes simplex virus-1 (HSV-1) Latency-associated transcript (LAT), can recruit Polycomb components to generate facultative heterochromatin during viral latency in neurons [28,29], and this may mimic mechanisms of telomeric heterochromatin formation.

Telomeres have been shown to have a complex 3D organizational structure that can affect chromatin and gene expression at subtelomeric sites [30,31,32]. Telomeres can cluster and localize at the nuclear periphery and nuclear pores [33]. Chromatin boundary factors, such as CCCTC-binding factor (CTCF) and cohesins, were found to bind to many subtelomeres to regulate TERRA expression and chromatin organization [34]. Many of these telomeric chromatin features are also found among DNA viruses that establish long-term persistent and latent infection, potentially reflecting shared mechanisms of chromatin organization with telomeres [35,36]. 

Telomere repeat length may also contribute to telomeric heterochromatin and affect local transcription through telomere DNA-looping into the subtelomeres [37]. Interestingly, subtelomeric genes regulated by telomere length are enriched for innate immune functions, such as Interferon-stimulated gene 15 (*ISG15*), an interferon-regulated gene that is among the most significantly activated upon viral infection. Thus, telomere repeat length and telomeric chromatin have functional links to innate immune signaling pathways, and cells with different telomere lengths may have different responses to viral infection. 

## 4. Viral Recapitulation of Telomere End-Protection

**Figure 1:**
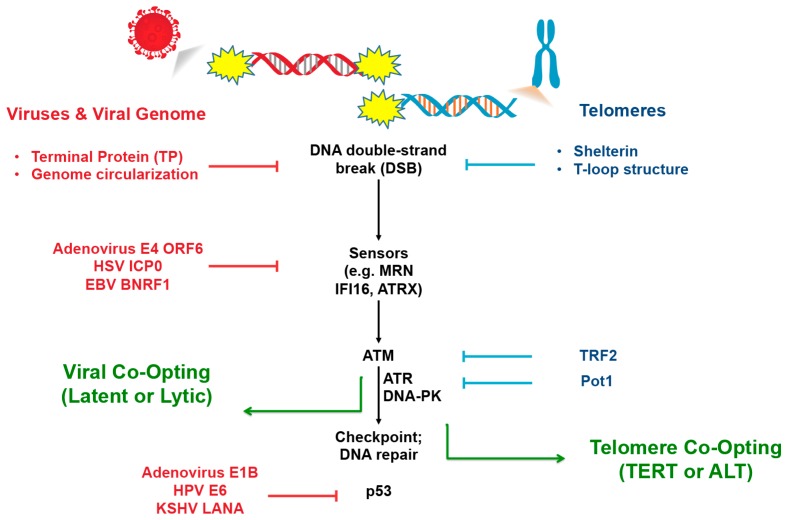
Strategies in suppressing or co-opting DNA Damage Response (DDR). DDR factors can suppress viral infection by inducing a checkpoint response culminating in p53 activation. Viral proteins, like adenovirus terminal protein (Ad TP) or E4 open reading frame (ORF)6, can prevent the DDR response at early stages, while other viral proteins block later events, such as the p53 function. Telomeric factors, such as TRF2 and Pot1, can also suppress DDR at telomeres. Both viruses and telomeres can co-opt DDR factors to replicate and maintain their genomes. TERT: telomerase protein subunit; ALT: alternative lengthening of telomeres; ATM: ataxia-telangectasia mutated; ATR: ATM-related; DNA-PK: DNA-protein kinase; MRN: MRE11-RAD50-NBS1 complex; HSV ICP0: herpes simplex 1 infected cell protein 0; EBV BNRF1:Epstein-Barr Virus BNRF1.

DNA virus infection in the nucleus can activate a DDR signaling pathway similar to that of a chromosome double strand break or an uncapped telomere [38]. Like telomeres, viruses have numerous mechanisms to evade the DDR, including the assembly of protective complexes at viral DNA ends that can actively suppress the cellular DDR (Figure 1). One example of a viral protein that protects the end of a viral genome is the adenovirus terminal protein (TP) that remains covalently linked to the end of the viral genome, and is necessary for viral DNA replication [39]. TP remains bound to adenovirus genomes upon entry, and may partially restrict some of the initial double strand break response and repair. However, TP is not sufficient to block the cellular DDR, as cellular repair enzymes, such as the MRE11-RAD50-NBS1 (MRN) complex, can remove TP to activate Ataxia-telangiectasia mutated protein (ATM) [40]. Additional adenovirus encoded proteins, including E4 open reading frame (ORF) 6, target MRE11 and NBS1 for ubiquitination and degradation to prevent this response, and evade the ligation and concatemerization of viral genomes that would otherwise block viral replication [40]. Thus, adenovirus, like telomeres, resists DNA-end-joining reactions that are catastrophic for genomic replication.

An alternative mechanism to evade double-strand break recognition is observed in herpesviruses that enter the nuclei as linear genomes, but then circularize at early time points of the viral life cycle [41,42,43]. Circularization can also be observed at some telomeres during ALT and may function as an alternative mechanism to protect and amplify telomere repeat DNA. At later stages of herpesvirus replication, the circularized viral genome provides a template for generating long head-to-tail concatemers of viral genomes via a rolling circle-based or recombination-based replication mechanism, potentially similar to what is observed in ALT [44]. HSV-1 replication activates ATM signaling and recruits several cellular homologous recombination factors (including MRN and Rad51) to the viral replication compartments [45,46]. Although herpesviruses activate ATM [47], the downstream DDR is attenuated by viral factors, such as infected cell protein 0 (ICP0), that degrade enzymes such as histone ubiquitin ligases RNF8 and RNF168 to prevent the formation of γH2AX and double strand break repair [48,49]. Concatemeric herpesvirus genomes are cleaved at their terminal repeats by a viral enzyme terminase, followed by the encapsidation into a preassembled capsid [50]. Analogously, telomere ends are processed by various exonucleases to enable the protective T-loop structures maintained by shelterin [51,52]. For most viruses, the replication process is further protected from cellular DDR through the formation of an elaborate viral replication compartment [53,54]. Viral replication compartments may provide a physical scaffold and barrier to protect viral genomes from host DDR. Similar super-structures are formed at telomeres during ALT and share many features with structures that form at viral ends during primary infection. 

**Figure 2:**
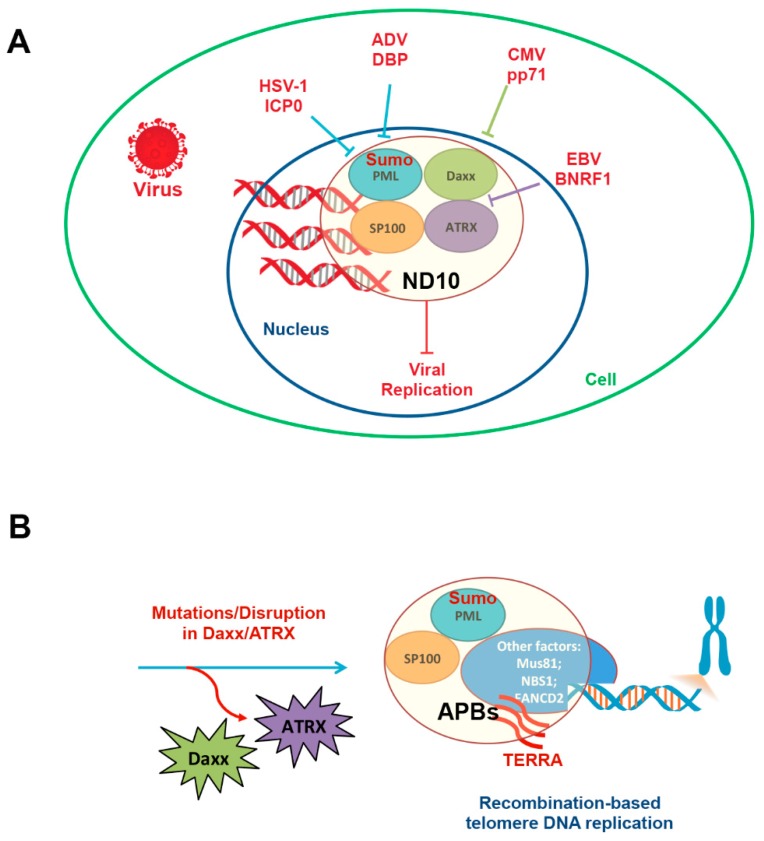
Role of PML-nuclear bodies (PML-NBs, or ND10) in regulating virus infection and telomere dysfunction. (**A**) PML-NBs can form at viral genomes to prevent viral infection. Viruses encode numerous factors that disable or degrade PML-NB components to enable viral infection or establishment of latency; (**B**) PML-NBs that have mutations in ATRX or Daxx can interact with telomeres to enable ALT. Telomere repeat-containing RNA (TERRA) is expressed at high-levels in ALT and during some viral infections. ADV DBP: DNA binding protein; APBs: ALT-associated PML-bodies; CMV: Cytomegalovirus; SUMO: small ubiquitin-like modifier.

Viral DNA is recognized by the promyelocytic leukemia-nuclear bodies (PML-NBs) (also referred to as ND10), an interferon-inducible nuclear structure consisting of core proteins PML, SP100, Daxx, and ATRX [55,56,57,58]. Numerous other proteins have been found to assemble at PML-NBs, including Heterochromatin Protein 1 (HP1), Topoisomerase II, and shelterin component TRF2, potentially depending on the cell-type and stress inducer (e.g., viral infection or telomere dysfunction). PML-NBs form in response to the incoming virus in the nucleus [59] and this process is partly dependent on the post-translational modification and recognition of the small ubiquitin-like modifier (SUMO) [60]. PML, a TRIM family member, is thought to possess E3 SUMO-ligase activity, while Daxx is known to have SUMO-interacting motifs (SIMs) that drive PML-NB formation [61]. DNA viruses disable or modify PML-NB components in various ways. HSV-1 ICP0 is a SUMO-targeted ubiquitin ligase (STUbL) that destabilizes PML to disassemble PML-NBs [62]. Adenovirus encoded DNA-binding protein (DBP) targets and modulates PML to assemble viral replication compartments [63]. CMV encodes a tegument protein, pp71, that degrades Daxx [64,65], while Epstein-Barr Virus (EBV) tegument protein BNRF1 dissociates ATRX from Daxx [66]. There are many other examples of virus disarming PML-NBs, consistent with this structure serving as a universal barrier to viral infection (Figure 2A).

PML-NBs have been implicated in the ALT [67,68,69]. However, ALT-associated PML-bodies (APBs) have distinct features from PML-NBs in non-ALT cells (Figure 2B). Typically, most ALT cells have genetic defects in ND10 components ATRX and Daxx [23,70,71]. Furthermore, APB assembly depends on various DNA damage proteins, including Mus81 [72], NBS1 [73] and FANCD2 [74]. Through small interfering (si)RNA screening and imaging analysis, APBs have been found to alter the chromatin structure and compaction of telomeres to elicit an ATM-dependent DNA damage response [75]. This process was dependent on SUMO1 and SUMO-E3 ligase MMS21 [76]. The DNA synthesis of telomeres in ALT involves a recombination-based mechanism, and more recent studies indicate that ATR-dependent [77], break-induced DNA synthesis plays a central role in this process [78]. Thus, ALT represents an alternative form of DNA replication and repair, which may be pirated by viruses during the viral infection cycle. Herpesvirus genomes circularize early after nuclear infection, and it is possible that ALT-like repair and recombination is required for this process. EBV has been shown to induce an ALT-like state in newly infected cells [79], so this would be consistent with a virus driving an existing cellular process to promote the viral life cycle. Herpesvirus replication involves a recombination-like process and the formation of replication centers that resemble cellular APBs [44]. HSV-1 undergoes complex rearrangements requiring host-cell recombination repair and synthesis [44,80], potentially utilizing a mechanism that is very similar to telomeric ALT. These findings indicate that there are remarkable similarities between cellular ALT and herpesvirus DNA virus replication. 

In addition to its anti-viral role and function in ALT, PML has been implicated in telomere maintenance in diploid fibroblasts [81]. The siRNA depletion of PML in a normal diploid fibroblast resulted in telomere damage, including end-to-end fusions, circle chromosomes, and fragile telomeres [81]. PML was found to associate with telomeres in normal cells, and this association increased with telomere attrition. Telomere-specific damage induced by G-quadruplex interacting agent RHPS4 increased the association of PML with telomeres [81]. These findings suggest that PML functions in both telomere surveillance, as well as in ALT and the intrinsic resistance to viral infection. 

Other cellular factors have been implicated in the intrinsic response to naked viral DNA in the nucleus. Among the first nuclear factors that recognize and respond to invading naked DNA is the Interferon-Inducible 16 (IFI16) protein, a member of the PYHIN family of DNA binding proteins ([82,83] reviewed in [84,85,86]). IFI16 signaling and function may be different for each virus. IFI16 is required for the maintenance of KSHV infection and is destroyed upon KSHV reactivation [87]. IFI16 remains chronically activated during EBV latency [88], but provides acute anti-viral signaling during HSV-1 infection [89,90,91]. The nucleation of IFI16 on DNA leads to its nuclear export and activation of the inflammasome, a cytoplasmic signaling cascade that activates nuclear factor κB (NFκB), interferon regulatory transcription factors (IRF) 3, and IRF7, and the activation of the interferon signaling pathways [92,93]. The mechanism of IFI16 binding and signaling in response to viral DNA in the nucleus has been reviewed extensively elsewhere [94]. Interestingly, IFI16 has been shown to bind to G-quadruplex DNA derived from human telomere repeats (TTAGGG)_n_, suggesting that it has the capacity to recognize telomeres [95]. However, it is not yet known whether IFI16 plays a role in recognizing uncapped telomeres in vivo.

## 5. Virus Infection Can Alter Telomere Maintenance

Virus infection can induce various cellular remodeling events and stress responses, including telomere-specific alterations. Several acute nuclear DNA viruses, including adenovirus, herpes simplex virus, cytomegalovirus, varicella zoster, and one RNA virus, influenza, were found to increase TERRA expression [96]. HSV-1 was unique among these for its rapid and robust activation of TERRA. This was further characterized and found to correlate with the capacity of HSV-1 to degrade PML-NBs through the activity of the ICP0 ring-domain E3 ubiquitin ligase activity. However, neither ICP0 by itself, nor PML depletion, was sufficient to induce TERRA, suggesting that additional viral factors and events are required to achieve the full activation of TERRA transcription. 

In addition to the activation of TERRA, HSV-1 infection produces numerous other changes in telomere maintenance. HSV-1 infection causes the dissociation of shelterin from telomeric DNA, the formation of single stranded DNA throughout telomeres, and the eventual degradation of telomeric repeats [96]. The HSV-1 encoded ICP8 protein, which has recA-like recombinase properties, was shown to associate with telomeric DNA and form pre-replication structures at telomeric foci. ICP8 was required for the formation of single-stranded telomeric DNA, and the subsequent loss of telomere repeat DNA at late stages of infection. However, like ICP0, the ectopic expression of ICP8 in the absence of other viral factors could not induce these changes at telomeres [96]. Thus, additional viral or cellular factors are required to collaborate with ICP8 to extensively remodel telomeres. These studies indicate that HSV-1 encodes multiple proteins that remodel telomeres to generate opportune sites for replication compartment assembly.

PML-NBs are thought to have both pro- and anti-viral features, depending on their composition and modifications [97,98]. PML-NBs can regulate herpesvirus entry and exit into latency. During HSV-1 latency in neurons, the formation of single viral-associated PML-NBs correlates with transcriptional silencing and the lack of detectable viral latency gene (LAT) expression [99]. However, when HSV-1 associates with multiple PML-NBs, LAT is expressed and the virus trends towards lytic reactivation. The latent-lytic switch of herpesviruses may be comparable with the cellular switch in telomere lengthening pathways. The exact mechanism that enables the switch from telomerase to the ALT pathway is not completely known, but appears to correlate with the loss of PML-NB components, especially ATRX and Daxx [23,70]. Thus, PML-NBs function dynamically to regulate both the HSV-1 latent-lytic switch [100], as well as the telomere replication-recombination switch. 

## 6. TERRA Induction by p53 Response Elements in Subtelomeres

**Figure 3:**
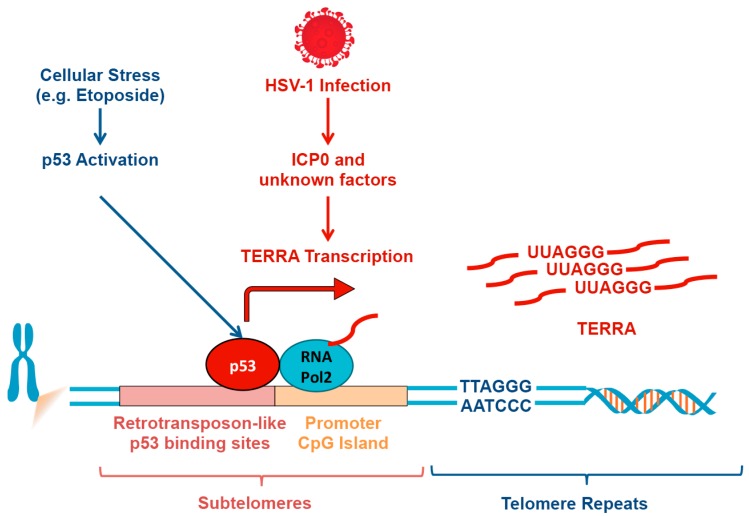
Induction of TERRA through subtelomeric retrotransposon-like p53 binding sites. Virus infection (e.g., HSV-1), as well as other cell stress pathways (e.g., DNA-damage, oxidative stress), can induce TERRA expression. Stress-associated p53 can bind subtelomeres to activate TERRA transcription. Subtelomeric p53 binding sites are evolutionarily derived from retrotransposon-like elements.

Telomeres are responsive to various stress response pathways, including viral infection [96], reactive oxygen species [16], and DNA damage signaling [101]. TERRA transcription can be induced by p53 activation and binding sites for p53 have been identified in human subtelomeres by ChIP-Seq (Figure 3). Among these were a series of repetitive elements containing p53 binding sites that occur in relatively close proximity (less than 10 kb) to the telomere repeat tract. These repetitive elements contain a non-canonical p53 binding site consisting of five half-sites, flanked by a GT-repeat track. These elements were further characterized as a telomere-specific LTR10b/MER61 family repeat element, a highly conserved and p53 consensus site-containing mobile element [102]. The LTR10b/MER61 repeat is found almost exclusively at or near telomere repeat tracks, and the one location far from the chromosome ends was found to be adjacent to an internal (interstitial) telomere repeat track. 

As a side note, the appearance of p53 binding sites in subtelomeric retrotransposon-like elements may reflect the evolutionary history of virus integration into telomeres. Retrotransposon insertion into subtelomeres is part of the natural mechanism of telomere length regulation in drosophila and other diptera [103,104]. Viral insertion in human subtelomeres may also be a frequent occurrence for some human viruses, including hepatitis B [105], and is reminiscent of the frequent integration of human herpesvirus 6 and 7, in addition to Marek’s disease virus (MDV), into telomere repeats (see other articles in this volume). 

The p53 binding sites in subtelomeres provide the transcriptional regulation of TERRA in response to various DNA damage and stress signaling pathways. Cells lacking p53 or the CRISPR engineered deletion of p53 binding sites in subtelomeres, led to a loss of TERRA expression and an increase in the accumulation of persistent γH2AX DNA damage signals at subtelomeres [106]. The loss of p53 also led to the instability of telomere repeats. These findings indicate that a retrotransposon-linked p53 binding site provides telomere repeat stability in human cells. They also highlight the evolutionary relationship between telomeres and viruses, including endogenous retrovirus-like elements that shape genomes. Additionally, the p53 binding sites at subtelomeres may also explain how telomeres and TERRA expression respond to the DNA-damage stress associated with a new viral infection. 

## 7. Antiviral Signaling Properties of TERRA

**Figure 4:**
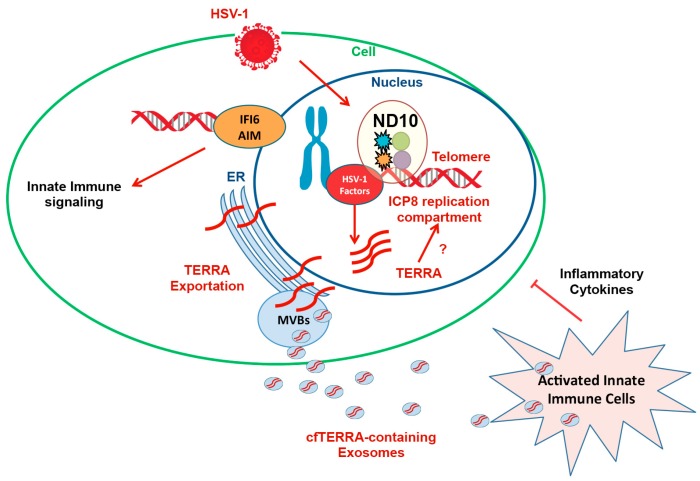
Innate immune response to viral infection or telomere dysfunction through the induction of TERRA. A small form of TERRA can be exported from the nucleus and cell through exosomes to stimulate inflammatory cytokine production in neighboring cells. Exosome-associated TERRA has viral-like properties including packaging and transmission. cfTERRA: Cell-free TERRA; IFI6: interferon-gamma induced protein 16; AIM2: Absent in Melanoma 2.

While TERRA can be induced to high levels in response to various stress, its potential function during viral infection remains mysterious. While the bulk of TERRA remains associated with telomeric DNA, similar to that observed in ALT cells, a small fraction of TERRA can be observed outside of the nucleus and in extracellular vesicles [107]. Further analysis revealed that a smaller form of TERRA can be transported from the nucleus and function in a signaling capacity (Figure 4). This smaller form of TERRA was enriched in exosome fractions, especially from lymphoblastoid cell lines that were latently infected with EBV [108].

Exosomes are nanosized vesicles (30–100 nm) secreted from cells to mediate intercellular communication and deliver functional factors [109]. Cell-free TERRA (cfTERRA) can be detected in tissue sections, cultured cell lines, and human blood plasma [108]. The levels of cfTERRA in exosomes reflect the status of telomeres [107]. During telomere dysfunction induced by the dominant negative TRF2 mutant, the fibroblast secreted more cfTERRA-containing exosomes [107]. Besides cfTERRA, the mRNA of telomerase human Telomerase Reverse Transcriptase (hTERT) was also shown to be secreted by tumor-derived exosomes [110]. These results highlight the previously unrecognized transport of telomeric factors.

The release of these telomeric factors has several impacts on the microenvironment. cfTERRA-containing exosomes were a strong inducer of inflammatory cytokines, including interleukin (IL)6 and tumor necrosis factor (TNF)α [108]. The increase in cfTERRA levels in response to dysfunctional telomeres was able to stimulate robust cytokine activation in naïve, neighboring cells. This cytokine activation suggests that cfTERRA-containing exosomes possess a telomere-associated molecular pattern (TAMP) that can trigger innate immune signaling [107]. Interestingly, exosomes from irradiated cells were shown to induce telomere shortening in the bystander cells in an RNA-dependent manner [111]. The mechanism for this phenomenon has not been elucidated, but it is possible that cfTERRA interferes with the telomere maintenance in the surrounding tissue. In addition, hTERT mRNA containing exosomes were able to transform nonmalignant fibroblasts into telomerase positive cells [107], indicating that exosomes have the potential to deliver tumor promoting factors. 

Viruses also hijack exosome signaling to evade host immunosurveillance. Cells latently infected with EBV type III latency had high levels of cfTERRA [108]. Type III latency cells express the viral latency membrane protein LMP1, which is known to promote exosome secretion [108]. Exosomes from EBV type III latency also contain EBV-encoded small RNA 1 (EBER1 and dUTPase, which can activate antiviral immunity from human plasmacytoid dendritic cells (pDCs) [112,113]. Virus infected cells may activate innate immune signaling of their neighbors through the exosome pathway. For example, HSV-1 infection induces the secretion of exosomes containing stimulator of interferon gene (STING), viral mRNAs, and microRNAs, which include all the components for stimulating the innate immune response in the recipient cells [114]. As HSV-1 infection induces TERRA transcription [96], HSV-1 infection is also likely to produce exosome-containing cfTERRA that contributes to strong innate immune signaling in the extracellular microenvironment. Similarly, hepatitis C virus (HCV) infection also displayed this restricted virulence through exosomes and innate immune signaling [115]. Whether exosome cargo serves the purpose of the virus or host may depend on the relative proportion of exosome content. 

Exosomes share several features with those of virus-like particles. Like viruses, exosomes are infectious particles containing protein and nucleic acids, but an important difference is that exosomes lack the capacity to replicate. However, their structural and functional similarity to viruses suggests that they share similar evolutionary origins. Viruses such as HIV-1 and the Hepatitis E virus utilize similar pathways as exosomes to facilitate viral infection and virion release [116,117]. Several viral vaccines have been developed by re-engineering the immunogenic properties of exosomes [118]. As cfTERRA is a highly stable and immunostimulatory molecule, it may provide an attractive adjuvant for exosome-based vaccine development. cfTERRA-containing exosomes may also serve as a useful biomarker for the detection of telomere dysfunction in the early stage of cancers or viral infection (Figure 4).

## 8. Conclusions

**Figure 5:**
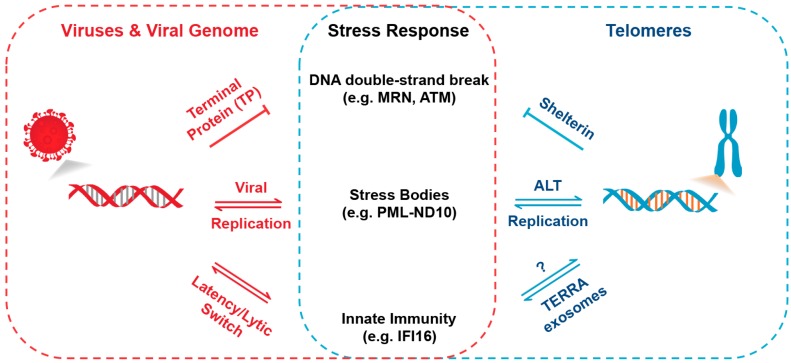
Common stress pathways regulating virus infection and telomeres. Comparison highlighting some common mechanisms used by viruses and telomeres to suppress and/or co-opt DDR and genome maintenance machinery.

The analogies between viruses and telomeres may be a useful framework to understand similar genetic and genomic processes, and act as a heuristic guide in developing new hypotheses to test experimentally or through bioinformatic forensics. Here, we have considered how viruses can resemble uncapped telomeric DNA, and share the strategies to evade DNA damage recognition and degradation. We have discussed the role of p53 response elements in subtelomeres and how these may affect telomeric adaptation to DNA damage stress, which is perhaps similar to how viruses may reactivate during latent infection. We have also considered how telomeres can express and package TERRA to communicate with neighboring cells, and how this resembles viral packaging and transmission. While some of the common mechanisms and themes are not limited to viruses or telomeres, and there is no surprise that viruses have captured many cellular processes, there remain compelling arguments that the common features of viruses and telomeres have emerged from either common ancestry or shared biological constraints (Figure 5). In either event, the comparative biology is likely to provide new perspectives and paradigms for the distinct disciplines of virology and telomere biology.
